# Prevalence of HTLV-1/2 in Pregnant Women Living in the Metropolitan Area of Rio de Janeiro

**DOI:** 10.1371/journal.pntd.0003146

**Published:** 2014-09-04

**Authors:** Denise Leite Maia Monteiro, Stella Regina Taquette, Danielle Bittencourt Sodré Barmpas, Nádia Cristina P. Rodrigues, Sérgio A. M. Teixeira, Lucia Helena C. Villela, Márcio Neves Bóia, Alexandre José Baptista Trajano

**Affiliations:** 1 Universidade do Estado do Rio de Janeiro (UERJ), Rio de Janeiro, Rio de Janeiro, Brazil; 2 Centro Universitário Serra dos Órgãos (UNIFESO), Teresópolis, Rio de Janeiro, Brazil; 3 Fundação Oswaldo Cruz (Fiocruz), Rio de Janeiro, Rio de Janeiro, Brazil; 4 Hospital Estadual da Mãe – Mesquita, Rio de Janeiro, Brazil; 5 Universidade do Grande Rio (UNIGRANRIO), Duque de Caxias, Rio de Janeiro, Brazil; University of Washington, United States of America

## Abstract

**Background:**

HTLV-1/2 infection can cause severe and disabling diseases in children and adults. The aim of the study was to estimate the prevalence of HTLV-1/2 infection in pregnant women living in the metropolitan area of Rio de Janeiro.

**Methodology/Principal Findings:**

1,204 pregnant women were tested upon hospital admission for delivery in two public hospitals in the cities of Rio de Janeiro and Mesquita, between November, 2012 and April, 2013. The samples were screened by chemiluminescent microparticle immunoassay (CMIA) and reactive ones were confirmed by Western blot (WB). Epi-info software was used for building the database and performing the statistical analysis. Eight patients had confirmed HTLV-1/2 infection (7 HTLV-1, one HTLV-2), equivalent to a prevalence rate of 0.66%. Two further reactive screening tests had negative Western blot results and therefore were considered negative in the statistical analysis. All HTLV-1/2-positive patients were born in Rio de Janeiro, most were non-Caucasian (87.5%), in a stable relationship (62.5%), had at least ten years of formal education (62.5%) and a monthly family income of up to US$600.00 (87.5%). There was only one case of coinfection with syphilis and none with HIV. The mean age of the infected women was 28.4 (SD = 6.3) years and of the seronegative ones was 24.8 (SD = 6.5) (p = 0.10). The median number of pregnancies were 3.0 and 1.0 (p = 0.06) and the median number of sexual partners were 3.5 and 3.0 (p = 0.33) in the seropositive and negative groups, respectively. There were no statistically significant differences between the groups.

**Conclusions/Significance:**

A significant prevalence of HTLV-1/2 was found in our population. The socio-epidemiological profile of carrier mothers was similar to the controls. Such findings expose the need for a public health policy of routine HTLV-1/2 screening in antenatal care, since counselling and preventive measures are the only strategies currently available to interrupt the chain of transmission and the future development of HTLV-1/2-related diseases.

## Author Summary

HTLV-1/2 are retroviruses transmitted by blood products, sexual contact and from mother to child, mainly through breastfeeding. The infection has a characteristic geographical distribution with endemic areas often neighbouring very low prevalence areas. Infection is life long and although asymptomatic in most cases, it can cause severe and disabling diseases in children and adults. There is currently no cure, vaccine or effective treatment for HTLV-1/2 infections. Our research is the first to study the prevalence of HTLV-1/2 in pregnant women living in the metropolitan area of Rio de Janeiro, the second largest in Brazil. 1,204 pregnant women were tested upon hospital admission for delivery in two public hospitals in the cities of Rio de Janeiro and Mesquita, between November, 2012 and April, 2013 and a significant prevalence of HTLV-1/2 was found (0.66%). The socio-epidemiological profile of carrier mothers was similar to the controls'. Epidemiological knowledge is fundamental for the elaboration of public health policies such as routine HTLV-1/2 screening in antenatal care, since counselling and preventive measures, mainly avoidance of breastfeeding, are the only strategies currently available to interrupt the chain of transmission and the future development of HTLV-1/2-related diseases.

## Introduction

Human T-lymphotropic virus (HTLV)-1 and HTLV-2 were the first oncogenic human retroviruses identified in the early 1980's [1]. They have a strong tropism for T-lymphocytes and are transmitted mostly via cell-cell infection [2].

HTLV-1/2 infection has a worldwide distribution, with an estimate of up to 15–20 million people affected [3]. Prevalence changes substantially according to geographical area, ethnical and social background and in specific risk groups such as intravenous (IV) drug users and sex workers [3]. HTLV-1 is highly prevalent in Sub-Saharan Africa, South-western Japan, Central and South America and in parts of the Middle East and Melanesia [3]. Regardless of the region, seroprevalence increases with age, particularly in women, in view of the excess efficiency of the male-female sexual transmission. HTLV-2 is endemic in several Native American populations and Pygmy tribes in Central Africa and also thrives in IV drug users worldwide, often in co-infection with HIV [4], [5].

Infection is life long and most of the patients remain asymptomatic, becoming viral reservoirs and keeping the chain of transmission. On the other hand, about 4% of HTLV-1 carriers will develop adult T-cell leukaemia/lymphoma (ATLL), a highly aggressive CD4^+^ T-cells malignancy. Type 1 virus is also associated with a wide range of inflammatory conditions, from the mild nonspecific dermatitis and uveitis to the disabling HTLV-1-associated myelopathy/Tropical Spastic Paraparesis (HAM/TSP) which affects 2–3% of the infected people [6]. HTLV-2 has been associated to hairy cell leukaemia, erithrodermatitis and a growing number of neurologic disorders [4]. Recent researches also suggest that the incidence of HTLV-1/2 linked diseases might be even higher than the literature traditionally reports due to the influence of local factors in their pathogenesis [7], [8].

There are three main routes of HTLV-1/2 transmission: sexual intercourse, infected whole blood or cell containing blood products and from mother-to-child (vertical transmission - VT). In endemic areas, VT remains the most important mode of transmission, since it occurs in up to 25% of the children who are breastfed by seropositive women [9]. Greater efficacy of VT is associated with the length of breastfeeding, high antibody titers and high proviral load in maternal blood and milk [10]–[12]. Intrauterine/perinatal transmission has been reported to have no significant epidemiological impact on disease burden [13], [14], however about 2.5% of the children of infected mothers will become HTLV-1/2-positive even in the absence of breastfeeding [7].

Given its continental dimensions, Brazil is thought to be one of the countries with the highest number of HTLV-1/2 carriers in the world. Estimates range widely from 800,000 to 2.5 million people [5], [15], [16]. Such discrepancy in numbers is a result of both the epidemiological characteristic of the infection, which has endemic clusters alongside low prevalence areas, and the gap in knowledge itself, since vast areas of the country remain unstudied.

Additionally, most of the research is done on potentially biased populations such as low risk voluntary blood donors or high risk IV drug users and sex clinic attendees. Intermediate seroprevalence rates have been found in pregnant women. Although not devoid of bias, this group has been proposed to represent a more reliable portrait of the general population since their prevalence data are generally able to characterize geographic areas likely to be endemic [3], [17].

Unfortunately there are no nationwide data on the seroprevalence of HTLV-1/2 in Brazilian pregnant women since screening is not recommended by the Ministry of Health as part of the routine antenatal care. Furthermore, there are no studies published about this prevalence on the State of Rio de Janeiro. Therefore the aim of this study was to estimate the prevalence of HTLV-1/2 infection in pregnant women admitted for delivery in the metropolitan area of Rio de Janeiro.

## Methods

### Study population

A total of 1,204 pregnant women were recruited upon admission for delivery in two public hospitals in the metropolitan area of Rio de Janeiro, between November 2012 and April 2013. The institutions involved in the study were ‘Pedro Ernesto’ University Hospital of the Rio de Janeiro State University (Universidade do Estado do Rio de Janeiro – HUPE/UERJ), a reference for high-risk obstetric patients located in the city of Rio de Janeiro, and ‘Hospital da Mãe’, a state hospital for low and medium-complexity obstetric care, in the city of Mesquita, metropolitan area of Rio de Janeiro. Thus, both low and high-risk obstetric populations were included. Patients who did not wish to take part in the research were excluded, as well as those considered mentally unable to give consent. At the enrolment, a structured questionnaire was applied by trained interviewers to collect epidemiological, social, clinical, gynaecological and obstetric data. HTLV-1/2 positive women were counseled by a multidisciplinary team which offered information about the infection and support. Breastfeeding was contraindicated and formula milk was provided to safeguard the infants' nutrition. All the children of carrier mothers are being prospectively followed by the Paediatrics Department of HUPE.

### Screening and confirmation tests

As per protocol of the Brazilian Ministry of Health all patients admitted for delivery have blood taken for HIV and Syphilis tests. At this moment, an extra sample was taken for HTLV-1/2 screening, which was performed by chemiluminescent microparticle immunoassay (CMIA - Architect rHTLV-I/II, Abbott). Cases of reactive serology were confirmed by Western blot (WB, Inno Lia HTLV-I/II score Biomerieux). All tests were carried out at the Clinical Analysis Laboratory of the HUPE/UERJ. The two patients with reactive screening tests and negative WB results were considered false positive and assigned to the negative group in the statistical analysis.

### Ethical aspects

This research complies with the ethical precepts established in the Declaration of Helsinki and the CONEP Resolution n° 196/96 and was approved by the Rio de Janeiro State University Research and Ethics Committee (COEP-UERJ, n.041/2012). Written informed consent was obtained from all the subjects and from the parents or guardians of the minors who agreed to take part. Anonymity and data confidentiality were guaranteed.

### Data analysis

The sample size of 1,188 patients was calculated considering an expected prevalence of 0.5% at a 95% confidence interval. Means, standard deviations, medians, interquartile ranges and percentages were used to describe the results. Medians were used to define the groups when numerical variables were analyzed as categorical ones (age, family income, number of partners and number of pregnancies). Missing data were excluded from the statistical analyses. Fisher Exact and Mann Whitney tests were used to assess the association between HTLV-1/2 infection and possible risk factors (demographic, social, familial, behavioral and sexual data). Logistic regression was performed to evaluate the relation between condom use and HTLV-1/2 occurrence adjusted by age and marital status. The EPI-INFO software version 3.5.2 was used for building the database and performing the statistical analyses.

## Results

Eight of the 1,204 patients had confirmed HTLV-1/2 infection (seven HTLV-1 and one HTLV-2) equivalent to a prevalence rate of 0.66%. Two further reactive screening tests had negative WB and were therefore considered false positive and excluded from the statistical analysis.

In the HTLV-1/2 positive group, all the patients were born on the State of Rio de Janeiro. They were mostly non-Caucasian (7/8 - 87.5%), reported being in a stable relationship (6/8 - 62.5%), had at least ten years of formal education (6/8 - 62.5%) and 87.5% declared having a monthly family income of up to US$600.00 (approximately two minimum wages). No women reported intravenous drug use, there was only one case of co-infection with syphilis and none with HIV.

The mean age of the infected pregnant women was 28.4 years (SD = 6.3) and of the seronegative ones was 24.8 (SD = 6.5) (p = 0.10). The mean age at first intercourse was approximately 16 years for both groups (μ = 15.9, SD = 1.9 *vs.* μ = 16.2, SD = 2.7). The median number of pregnancies were 3 and 1 (p = 0.06) and the median number of sexual partners were 3.5 and 3.0 (p = 0.33) in the seropositive and negative patients, respectively.

The comparison between the HTLV-1/2-positive and negative groups found no statistically significant difference for any of the variables assessed: age, ethnicity, marital status, family income, level of education, age at first intercourse, number of partners, number of pregnancies, use of condom or co-infection with syphilis or HIV (Table 1).

**Table 1:** Analysis for socio-demographic and sexual variables in HTLV-1/2 positive and negative pregnant women.

	Category	HTLV-1/2+	HTLV-1/2−	p-value
		n (%)	n (%)	
**Age (years)**	<24	2 (25.0)	646 (54.2)	0.10
	≥25	6 (75.0)	546 (45.8)	
**Ethnicity**	caucasian	1 (12.5)	351 (29.4)	
	non caucasian	7 (87.5)	841 (70.6)	0.27
**Marital status**	married/partnership	5 (62.5)	883 (73.9)	
	others	3 (37.5)	312 (26.1)	0.35
**Family income (MW)**	≤2	7 (87.5)	992 (83.6)	
	>2	1 (12.5)	195 (16.4)	0.61
**Education (years)**	<10	3 (37.5)	425 (35.5)	
	≥10	5 (62.5)	767 (64.1)	0.59
**First intercourse (years)**	≤16	3 (37.5)	555 (46.4)	
	>16	5 (62.5)	641 (53.6)	0.45
**Number of partners**	≤3	4 (50.0)	789 (69.6)	
	>3	4 (50.0)	345 (30.4)	0.20
**Number of pregnancies**	≤2	3 (37.5)	728 (69.7)	
	>2	5 (62.5)	317 (30.3)	0.06
**Condom use**	yes	3 (37.5)	718 (60.1)	
	no	5 (62.5)	477 (39.9)	0.17
**HIV/Syphilis**	yes	1 (12.5)	65 (5.4)	
	no	7 (87.5)	1,131 (94.6)	0.36

MW – minimum wages. Missing data were excluded from the analysis.

After controlling the association between seropositivity and the number of previous pregnancies by marital status and condom use it was observed that women with two or more previous gestations were about three times more likely to be infected (OR = 3.63). However only a borderline statistic significance was found (p = 0.08).

## Discussion

Antenatal groups are considered to better represent the general population than blood donors and IV drug users, being of particular interest for the chain of transmission since most of the vertical transmission can be prevented by avoiding breastfeeding.

A review of the literature retrieved no published data on the prevalence of HTLV-1/2 infection in pregnant women in the State of Rio de Janeiro. The Brazilian articles concerning the infection display a significant geographic heterogeneity in prevalence, ranging from 0.07% on the State of Minas Gerais [18] to around 1% in the State of Bahia [19]–[21] (Table 2). Our research found a prevalence of 0.66% which would place the metropolitan area of Rio de Janeiro as the second highest seroprevalence in pregnant women in Brazil. This number is 40% higher than it was found in blood donors (0.47%) on the same state in a research performed between 1995 and 2000 [22]. However, this comparison is likely to be inaccurate in view of the long time elapsed between the studies. In Europe, HTLV-1/2 prevalence was found to be 5–10 times higher in pregnant women than in blood donors, regardless of the area specific prevalence [17].

**Table 2:** Seroepidemiology of HTLV-1/2 in Brazil during the pregnancy and puerperium.

Authors/Year	City, State	n	Prevalence rate (%)
Broutet *et al*, 1996 [33]	Fortaleza, CE†	814	0.12
Bittencourt *et al*, 2001 [19]	Salvador, BA†	6,754	0.84
Olbrich Neto *et al*, 2004 [29]	Botucatu, SP†	913	0.10
Oliveira *et al*, 2006 [26]	GO†	15,485	0.10
Figueiró-Filho *et al*, 2007 [34]	MS†	32,512	0.13
Dal Fabbro *et al*, 2008 [25]	MS	116,689	0.13
Magalhães *et al*, 2008 [20]	Cruz das Almas, BA	408	0.98
Ydy *et al*, 2009 [35]	Cuiabá, MT†	2,965	0.20
Lima *et al*, 2009 [27]	Vitória, ES†	534	0.60*
Ribeiro *et al*, 2010 [18]	MG†	55,293	0.07
Sequeira *et al*, 2012 [30]	PA†	13,382	0.30
Guimarães Souza *et al*, 2012 [15]	São Luís, MA†	2,044	0.30
Mello *et al*, 2014 [21]	Ilhéus/Itabuna, BA	2,766	1.05

* ELISA results unconfirmed by WB.

† CE: Ceará; BA: Bahia; SP: São Paulo; GO: Goiás; MS: Mato Grosso do Sul; MT: Mato Grosso; ES: Espírito Santo; MG: Minas Gerais; PA: Pará; MA: Maranhão.

A comprehensive Brazilian study performed at the Public Blood Center Network comprising over 6 million blood donations from all the 27 state capitals revealed a striking geographic variability of the HTLV-1/2 infection [22]. It ranged from 0.4/1,000 in Florianópolis (Santa Catarina State) to a rate 25 times higher (10.0/1,000) in São Luís (Maranhão State). It's noteworthy that likewise in pregnant women, the Brazilian states with the highest seroprevalence were located on the North and Northeast of the country (Pará, Pernambuco, Bahia and Maranhão) while prevalence rates were markedly lower in the main cities of the Southern states, such as Santa Catarina [22]. These findings were corroborated by a more recent research in blood donors of three densely populated state capitals (Pernambuco, Minas Gerais and São Paulo) [23].

The regional clustering phenomenon can be explained by the influence of the ethnical characteristics of the population in the prevalence of HTLV-1/2. The northern area of Brazil has a greater proportion of African and native-descendants than the South. There is strong evidence of endemicity of HTLV-2 among groups who originally inhabited the Amazon [5]. Additionally, there is abundant phylogenetic evidence of the introduction of HTLV-1 strains via the forced migrations from the African continent [20], [24] which is thought to be the place of origin of human retroviruses and the largest endemic area for HTLV-1 in the world. Nevertheless even in Africa the seroprevalence for HTLV-1/2 in pregnant women can vary widely from 5.5% in Nigeria to 0.2% in South Africa, with places of intermediate prevalence such as the Republic of Congo (0.7% - similar to our own) [3].

A strength of the present study is that all HTLV-1/2-positive subjects were born in the researched area, confirming it as a portrait of the local population. The mean age of positive pregnant women and the fact that they were mostly non-Caucasian are in accordance with the literature [3], [15], [19], [23], [25]. In the comparison between HTLV-1/2 positive and negative groups none of the variables was statistically significant, which was in line with the two latest studies from Bahia [20], [21]. Although there were some reports of an inverse association between both length of formal education and family income with HTLV-1/2 infection [7], [18], [19], [23]–[26] these were not universal findings [15], [21], [27]–[30] and were not confirmed in our population.

The similarity of the epidemiological profile in both of our groups and the lack of clear risk factors seem to point to routine HTLV-1/2 prenatal screening as the most sensible path to follow, as supported by other colleagues [8], [16], [18], [20], [21], [25], [28], [30], [31]. Once carrier mothers were identified, avoidance of breastfeeding would be a fundamental tool for the control of mother-to-child transmission. Taking in consideration the number of live births in the metropolitan area of Rio de Janeiro (135,938 in 2013) and the seroprevalence observed in our study, the introduction of routine HTLV-1/2 antenatal screening could avoid up to 900 cases of vertical transmission per year [32].

A limitation of our research was the possibility of selection bias. Patients were recruited at admission for delivery, which could have underestimated the overall prevalence in pregnancy since we might have lost the patients who miscarried and those who eventually had preterm labour or stillbirths.

Implications for future research – our finding of such a significant prevalence shows the need for studies on a larger scale, testing all pregnant women at the beginning of antenatal care in order to avoid selection bias and increase the confidence in our results. Sampling a broader population can help to determine the cost-effectiveness of the test and justify its introduction to routine prenatal screening. Following the children of infected mothers in a cohort study could identify cases of vertical transmission and the incidence of HTLV-1/2 related diseases in our population.

Universal screening for HTLV-1/2 in blood donors was implemented in Brazil in 1993 with significant impact on this mode of transmission. Additionally, horizontal transmission is thought to have been greatly reduced by successful public policies aiming at the prevention of other sexually transmitted diseases. Since 2002 HIV and syphilis prenatal screening tests are mandatory according to the Brazilian Ministry of Health in order to prevent mother-to-child transmission. HTLV screening was not included in this guideline; in fact it's not even listed as an infectious disease worthy of being targeted by government policies [31]. Therefore VT of HTLV-1/2 remains the only mode of transmission unaddressed by public health policies in our country.

Since currently there is no cure, effective treatment or immunization for HTLV-1/2 infection and its complications, more accurate knowledge about its prevalence is helpful in the elaboration of public policies on educational and prophylactic measures to increase awareness and reduce the rates of viral transmission and the incidence of infection-related diseases.

## Supporting Information

Supporting Information S1 STROBE Checklist.
(PDF)

Supporting Information S2 Data file.
(XLS)

Supporting Information S3 Statistical analysis.
(PDF)
