# Supplementary material for: Prevalence of HTLV-1/2 in Pregnant Women Living in the Metropolitan Area of Rio de Janeiro
Source: PLoS Negl Trop Dis. 2014 Sep 4;8(9):e3146. doi: 10.1371/journal.pntd.0003146 (PMC4154655; doi:10.1371/journal.pntd.0003146)
Supplement: Supporting Information S3 — Statistical analysis. (PDF) [file pntd.0003146.s003.pdf]

Epi Info

Results Library

CurrentView:	**C:\Users\nadia.rodrigues\Dropbox\pesquisas2014\Dani\bancos\Projeto ApoioHU atualizado~24122013.MDB:viewPRINCIPAL**
Relate:	**viewIDADOSSOCIODEMOGRÁFICOS**
Relate:	**viewRESULTADOEXAMESHUPE**
Record Count:	**1204** (Deletedrecordsexcluded)

FREQ HTLVHUPE

Next Procedure

Forward

HTLV	Frequency	Percent	Cum Percent	
**Yes**	8	0.7%	0.7%	
**No**	1196	99.3%	100.0%	
**Total**	1204	100.0%	100.0%	

95% ConfLimits

Yes 0.3% 1.4%

No 98.6% 99.7%

PreviousDataset Results Library

FREQ ETNIA

Next Procedure

Forward

ETNIA / COR	Frequency	Percent	Cum Percent	
**1**	352	29.3%	29.3%	
**2**	242	20.2%	49.5%	
**3**	598	49.8%	99.3%	
**4**	8	0.7%	100.0%	
**Total**	1200	100.0%	100.0%	

95% ConfLimits

1 26.8% 32.0%

2 18.0% 22.6%

3 47.0% 52.7%

4 0.3% 1.4%

PreviousDataset Results Library

FREQ RENDAFAMMENS

Next Procedure

Forward

RENDA FAMILIAR MENSAL ESTIMADA (EM SALÁRIOS MÍNIMOS)	Frequency	Percent	Cum Percent	
**0**	2	0.2%	0.2%	
**1**	638	53.4%	53.6%	
**2**	359	30.0%	83.6%	
**3**	122	10.2%	93.8%	
**4**	38	3.2%	97.0%	
**5**	14	1.2%	98.2%	
**6**	22	1.8%	100.0%	
**Total**	1195	100.0%	100.0%	

95%	ConfLimits	
0	0.0%	0.7%
1	50.5%	56.2%
2	27.5%	32.7%
3	8.6%	12.1%
4	2.3%	4.4%
5	0.7%	2.0%
6	1.2%	2.8%

PreviousDataset Results Library

FREQ ESCOLARIDADE

Next Procedure

Forward

ESCOLARIDADE	Frequency	Percent	Cum Percent	
**1**	3	0.3%	0.3%	
**2**	83	6.9%	7.2%	
**3**	223	18.6%	25.8%	
**4**	119	9.9%	35.7%	
**5**	255	21.3%	56.9%	
**6**	436	36.3%	93.3%	
**7**	81	6.8%	100.0%	
**Total**	1200	100.0%	100.0%	

95% ConfLimits

1	0.1%	0.8%
2	5.6%	8.5%
3	16.4%	20.9%
4	8.3%	11.8%
5	19.0%	23.7%
6	33.6%	39.1%
7	5.4%	8.4%

PreviousDataset Results Library

FREQ ESTADOCONJUGAL

Next Procedure

Forward

ESTADO CONJUGAL	Frequency	Percent	Cum Percent	
**1**	888	73.8%	73.8%	
**2**	298	24.8%	98.6%	
**3**	16	1.3%	99.9%	
**4**	1	0.1%	100.0%	
**Total**	1203	100.0%	100.0%	

95% ConfLimits

1	71.2%	76.3%
2	22.4%	27.3%
3	0.8%	2.2%
4	0.0%	0.5%

PreviousDataset Results Library

MEANS NUMPARCSEXVIDA HTLVHUPE

Next Procedure

Forward

HTLV			
**NÚM DE PARCEIROS ATÉ O MOMENTO**	**Yes**	**No**	**TOTAL**
**1**	1	325	326
Row %	0.3	99.7	100.0
Col %	12.5	27.6	27.5
**2**	2	229	231
Row %	0.9	99.1	100.0
Col %	25.0	19.4	19.5
**3**	1	235	236
Row %	0.4	99.6	100.0
Col %	12.5	19.9	19.9
**4**	1	136	137
Row %	0.7	99.3	100.0
Col %	12.5	11.5	11.5
**5**	2	100	102
Row %	2.0	98.0	100.0
Col %	25.0	8.5	8.6
**6**	0	38	38
Row %	0.0	100.0	100.0
Col %	0.0	3.2	3.2
**7**	0	15	15
Row %	0.0	100.0	100.0
Col %	0.0	1.3	1.3
**8**	0	17	17
Row %	0.0	100.0	100.0

Col %	0.0	1.4	1.4
**9**<br>Row %<br>Col %	0<br>0.0<br>0.0	6<br>100.0<br>0.5	6<br>100.0<br>0.5
**10**<br>Row %<br>Col %	1<br>2.9<br>12.5	33<br>97.1<br>2.8	34<br>100.0<br>2.9
**11**<br>Row %<br>Col %	0<br>0.0<br>0.0	2<br>100.0<br>0.2	2<br>100.0<br>0.2
**12**<br>Row %<br>Col %	0<br>0.0<br>0.0	4<br>100.0<br>0.3	4<br>100.0<br>0.3
**13**<br>Row %<br>Col %	0<br>0.0<br>0.0	2<br>100.0<br>0.2	2<br>100.0<br>0.2
**14**<br>Row %<br>Col %	0<br>0.0<br>0.0	1<br>100.0<br>0.1	1<br>100.0<br>0.1
**15**<br>Row %<br>Col %	0<br>0.0<br>0.0	8<br>100.0<br>0.7	8<br>100.0<br>0.7
**20**<br>Row %<br>Col %	0<br>0.0<br>0.0	10<br>100.0<br>0.8	10<br>100.0<br>0.8
**25**<br>Row %	0<br>0.0	2<br>100.0	2<br>100.0

Col %	0.0	0.2	0.2
**30**	0	5	5
Row %	0.0	100.0	100.0
Col %	0.0	0.4	0.4
**34**	0	1	1
Row %	0.0	100.0	100.0
Col %	0.0	0.1	0.1
**37**	0	1	1
Row %	0.0	100.0	100.0
Col %	0.0	0.1	0.1
**40**	0	1	1
Row %	0.0	100.0	100.0
Col %	0.0	0.1	0.1
**50**	0	3	3
Row %	0.0	100.0	100.0
Col %	0.0	0.3	0.3
**99**	0	5	5
Row %	0.0	100.0	100.0
Col %	0.0	0.4	0.4
**TOTAL**	8	1179	1187
Row %	0.7	99.3	100.0
Col %	100.0	100.0	100.0

Descriptive Statistics for Each Value of Crosstab Variable

Obs	Total	Mean	Variance	StdDev

Yes	8	32.0000	4.0000	8.0000	2.8284
No	1179	4771.0000	4.0466	58.7729	7.6663

	Minimum	25%	Median	75%	Maximum	Mode
Yes	1.0000	2.0000	3.5000	5.0000	10.0000	2.0000
No	1.0000	1.0000	3.0000	4.0000	99.0000	1.0000

ANOVA, a Parametric Test for Inequality of Population Means

(For normally distributed data only)

Variation	SS	df	MS	F statistic
Between	0.0173	1	0.0173	0.0003
Within	69290.4343	1185	58.4729	
Total	69290.4516	1186		

T Statistic = 0.0172

P-value = 0.9863

Bartlett's Test for Inequality of Population Variances

Bartlett's chi square= 7.5380 df=1 P value=0.0060

A small p-value (e.g., less than 0.05 suggests that the variances are not homogeneous and that the ANOVA may not be appropriate.

Mann-Whitney/Wilcoxon Two-Sample Test (Kruskal-Wallis test for two groups)

Kruskal-Wallis H (equivalent to Chi square) = .9455
Degreesoffreedom = 1
P value = 0.3309

PreviousDataset Results Library

FREQ USOPRESERVANTESGRAV

Next Procedure

Forward

USO DE PRESERVATIVO ANTES DA GRAVIDEZ	Frequency	Percent	Cum Percent	
**1**	200	16.6%	16.6%	
**2**	521	43.3%	59.9%	
**3**	482	40.1%	100.0%	
**Total**	1203	100.0%	100.0%	

95% ConfLimits

1 14.6% 18.9%
2 40.5% 46.2%
3 37.3% 42.9%

MEANS Gesta HTLVHUPE

Next Procedure

Forward

HTLVHUPE			
**Gesta**	**Yes**	**No**	**TOTAL**
**1**	2	453	455
Row %	0,4	99,6	100,0
Col %	25,0	43,3	43,2
**2**	1	275	276
Row %	0,4	99,6	100,0
Col %	12,5	26,3	26,2
**3**	2	146	148
Row %	1,4	98,6	100,0
Col %	25,0	14,0	14,0
**4**	2	97	99
Row %	2,0	98,0	100,0
Col %	25,0	9,3	9,4
**5**	1	36	37
Row %	2,7	97,3	100,0
Col %	12,5	3,4	3,5
**6**	0	19	19
Row %	0,0	100,0	100,0
Col %	0,0	1,8	1,8

**7**	0	8	8
Row %	0,0	100,0	100,0
Col %	0,0	0,8	0,8
**8**	0	3	3
Row %	0,0	100,0	100,0
Col %	0,0	0,3	0,3
**9**	0	4	4
Row %	0,0	100,0	100,0
Col %	0,0	0,4	0,4
**10**	0	4	4
Row %	0,0	100,0	100,0
Col %	0,0	0,4	0,4
**11**	0	1	1
Row %	0,0	100,0	100,0
Col %	0,0	0,1	0,1
**TOTAL**	8	1046	1054
Row %	0,8	99,2	100,0
Col %	100,0	100,0	100,0

Descriptive Statistics for Each Value of Crosstab Variable

	Obs	Total	Mean	Variance	Std Dev
Yes	8	23,0000	2,8750	2,1250	1,4577
No	1046	2290,0000	2,1893	2,3144	1,5213

	Minimum	25%	Median	75%	Maximum	Mode
Yes	1,0000	1,5000	3,0000	4,0000	5,0000	1,0000

No	1,0000	1,0000	2,0000	3,0000	11,0000	1,0000

ANOVA, a Parametric Test for Inequality of Population Means

(For normally distributed data only)

Variation	SS	df	MS	F statistic
Between	3,7330	1	3,7330	1,6138
Within	2433,3951	1052	2,3131	
Total	2437,1281	1053		

T Statistic = 1,2704

P-value = 0,2040

Bartlett's Test for Inequality of Population Variances

Bartlett's chi square= 0,0235 df=1 P value=0,8781

A small p-value (e.g., less than 0.05 suggests that the variances are not homogeneous and that the ANOVA may not be appropriate.

Mann-Whitney/Wilcoxon Two-Sample Test (Kruskal-Wallis test for two groups)

Kruskal-Wallis H (equivalent to Chi square) = 2,5406
Degrees of freedom = 1

P value = 0,1110

PreviousDataset Results Library

MEANS IDADE HTLVHUPE

Next Procedure

Forward

	HTLV		
**IDADE**	**Yes**	**No**	**TOTAL**
**13**	0	1	1
Row %	0.0	100.0	100.0
Col %	0.0	0.1	0.1
**14**	0	5	5
Row %	0.0	100.0	100.0
Col %	0.0	0.4	0.4
**15**	0	21	21
Row %	0.0	100.0	100.0
Col %	0.0	1.8	1.8
**16**	0	40	40
Row %	0.0	100.0	100.0
Col %	0.0	3.4	3.3
**17**	0	60	60

Row %	0.0	100.0	100.0
Col %	0.0	5.0	5.0
**18**	1	82	83
Row %	1.2	98.8	100.0
Col %	12.5	6.9	6.9
**19**	0	80	80
Row %	0.0	100.0	100.0
Col %	0.0	6.7	6.7
**20**	0	82	82
Row %	0.0	100.0	100.0
Col %	0.0	6.9	6.8
**21**	1	73	74
Row %	1.4	98.6	100.0
Col %	12.5	6.1	6.2
**22**	0	73	73
Row %	0.0	100.0	100.0
Col %	0.0	6.1	6.1
**23**	0	68	68
Row %	0.0	100.0	100.0
Col %	0.0	5.7	5.7
**24**	0	61	61
Row %	0.0	100.0	100.0
Col %	0.0	5.1	5.1
**25**	0	73	73
Row %	0.0	100.0	100.0
Col %	0.0	6.1	6.1
**26**	1	50	51

Row %<br>Col %	2.0<br>12.5	98.0<br>4.2	100.0<br>4.3
**27**<br>Row %<br>Col %	0<br>0.0<br>0.0	54<br>100.0<br>4.5	54<br>100.0<br>4.5
**28**<br>Row %<br>Col %	0<br>0.0<br>0.0	44<br>100.0<br>3.7	44<br>100.0<br>3.7
**29**<br>Row %<br>Col %	1<br>2.6<br>12.5	37<br>97.4<br>3.1	38<br>100.0<br>3.2
**30**<br>Row %<br>Col %	1<br>2.2<br>12.5	44<br>97.8<br>3.7	45<br>100.0<br>3.8
**31**<br>Row %<br>Col %	0<br>0.0<br>0.0	41<br>100.0<br>3.4	41<br>100.0<br>3.4
**32**<br>Row %<br>Col %	0<br>0.0<br>0.0	35<br>100.0<br>2.9	35<br>100.0<br>2.9
**33**<br>Row %<br>Col %	1<br>5.0<br>12.5	19<br>95.0<br>1.6	20<br>100.0<br>1.7
**34**<br>Row %<br>Col %	0<br>0.0<br>0.0	26<br>100.0<br>2.2	26<br>100.0<br>2.2
**35**	2	26	28

Row %<br>Col %	7.1<br>25.0	92.9<br>2.2	100.0<br>2.3
**36**<br>Row %<br>Col %	0<br>0.0<br>0.0	19<br>100.0<br>1.6	19<br>100.0<br>1.6
**37**<br>Row %<br>Col %	0<br>0.0<br>0.0	21<br>100.0<br>1.8	21<br>100.0<br>1.8
**38**<br>Row %<br>Col %	0<br>0.0<br>0.0	18<br>100.0<br>1.5	18<br>100.0<br>1.5
**39**<br>Row %<br>Col %	0<br>0.0<br>0.0	12<br>100.0<br>1.0	12<br>100.0<br>1.0
**40**<br>Row %<br>Col %	0<br>0.0<br>0.0	12<br>100.0<br>1.0	12<br>100.0<br>1.0
**41**<br>Row %<br>Col %	0<br>0.0<br>0.0	8<br>100.0<br>0.7	8<br>100.0<br>0.7
**42**<br>Row %<br>Col %	0<br>0.0<br>0.0	3<br>100.0<br>0.3	3<br>100.0<br>0.3
**43**<br>Row %<br>Col %	0<br>0.0<br>0.0	3<br>100.0<br>0.3	3<br>100.0<br>0.3
**44**	0	1	1

Row %	0.0	100.0	100.0
Col %	0.0	0.1	0.1
**TOTAL**	8	1192	1200
Row %	0.7	99.3	100.0
Col %	100.0	100.0	100.0

Descriptive Statistics for Each Value of Crosstab Variable

	Obs	Total	Mean	Variance	StdDev
Yes	8	227.0000	28.3750	39.9821	6.3231
No	1192	29601.0000	24.8331	42.0939	6.4880

	Minimum	25%	Median	75%	Maximum	Mode
Yes	18.0000	23.5000	29.5000	34.0000	35.0000	35.0000
No	13.0000	20.0000	24.0000	29.0000	44.0000	18.0000

ANOVA, a Parametric Test for Inequality of Population Means

(For normally distributed data only)

Variation	SS	df	MS	F statistic
Between	99.6940	1	99.6940	2.3691
Within	50413.6527	1198	42.0815	
Total	50513.3467	1199		

T Statistic = 1.5392

P-value = 0.1238

Bartlett's Test for Inequality of Population Variances

Bartlett's chi square= 0.0087 df=1 P value=0.9259

A small p-value (e.g., less than 0.05 suggests that the variances are not homogeneous and that the ANOVA may not be appropriate.

Mann-Whitney/Wilcoxon Two-Sample Test (Kruskal-Wallis test for two groups)

Kruskal-Wallis H (equivalent to Chi square) = 2.5580
Degreesoffreedom = 1
P value = 0.1097

TABLES HTLVHUPE IDADE_cod

Next Procedure

Forward

IDADE_COD			
**HTLVHUPE**	**1**	**2**	**TOTAL**

**Yes**	2	6	8
Row %	25,0	75,0	100,0
Col %	0,3	1,1	0,7
**No**	646	546	1192
Row %	54,2	45,8	100,0
Col %	99,7	98,9	99,3
**TOTAL**	648	552	1200
Row %	54,0	46,0	100,0
Col %	100,0	100,0	100,0

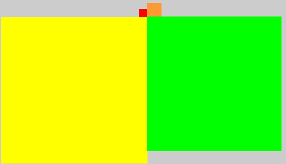

Single Table Analysis

	Point	95% Confidence Interval		
	Estimate	Lower	Upper	
PARAMETERS: Odds-based				
Odds Ratio (cross product)	0,2817	0,0566	1,4016	(T)
Odds Ratio (MLE)	0,2820	0,0390	1,3384	(M)
		0,0277	1,5853	(F)
PARAMETERS: Risk-based				
Risk Ratio (RR)	0,4613	0,1387	1,5337	(T)
Risk Difference (RD%)	-29,1946	-59,3339	0,9446	(T)

(T=Taylor series; C=Cornfield; M=Mid-P; F=Fisher Exact)

STATISTICAL TESTS	Chi-square	1-tailed p	2-tailed p

Chi-square - uncorrected	2,7267		0,0986832673
Chi-square - Mantel-Haenszel	2,7244		0,0988238032
Chi-square - corrected (Yates)	1,6781		0,1951840404
Mid-p exact		0,0589692892	
Fisher exact		0,0974378258	

Warning: The expected value of a cell is <5. Fisher Exact Test should be used.

PreviousDataset Results Library

MEANS IDADEINICIOSEXO

Next Procedure

Forward

IDADE (EM ANOS) DE INÍCIO DA ATIVIDADE SEXUAL	Frequency	Percent	Cum Percent	
**7**	1	0.1%	0.1%	
**8**	1	0.1%	0.2%	
**9**	1	0.1%	0.2%	
**10**	2	0.2%	0.4%	
**11**	5	0.4%	0.8%	
**12**	31	2.6%	3.4%	
**13**	92	7.6%	11.0%	

**14**	187	15.5%	26.6%	
**15**	238	19.8%	46.3%	
**16**	199	16.5%	62.9%	
**17**	155	12.9%	75.7%	
**18**	131	10.9%	86.6%	
**19**	56	4.7%	91.3%	
**20**	28	2.3%	93.6%	
**21**	25	2.1%	95.7%	
**22**	12	1.0%	96.7%	
**23**	11	0.9%	97.6%	
**24**	6	0.5%	98.1%	
**25**	10	0.8%	98.9%	
**26**	7	0.6%	99.5%	
**27**	3	0.2%	99.8%	
**32**	2	0.2%	99.9%	
**33**	1	0.1%	100.0%	
**Total**	1204	100.0%	100.0%	

Obs	Total	Mean	Variance	StdDev
1204	19452.0000	16.1561	7.4453	2.7286

Minimum	25%	Median	75%	Maximum	Mode
7.0000	14.0000	16.0000	17.0000	33.0000	15.0000

PreviousDataset Results Library

MEANS IDADEINICIOSEXO HTLVHUPE

Next Procedure

Forward

HTLV			
**IDADE (EM ANOS) DE INÍCIO DA ATIVIDADE SEXUAL**	**Yes**	**No**	**TOTAL**
**7**	0	1	1
Row %	0.0	100.0	100.0
Col %	0.0	0.1	0.1
**8**	0	1	1
Row %	0.0	100.0	100.0
Col %	0.0	0.1	0.1
**9**	0	1	1
Row %	0.0	100.0	100.0
Col %	0.0	0.1	0.1
**10**	0	2	2
Row %	0.0	100.0	100.0
Col %	0.0	0.2	0.2
**11**	0	5	5
Row %	0.0	100.0	100.0
Col %	0.0	0.4	0.4
**12**	0	31	31
Row %	0.0	100.0	100.0
Col %	0.0	2.6	2.6
**13**	1	91	92

Row %<br>Col %	1.1<br>12.5	98.9<br>7.6	100.0<br>7.6
**14**<br>Row %<br>Col %	1<br>0.5<br>12.5	186<br>99.5<br>15.6	187<br>100.0<br>15.5
**15**<br>Row %<br>Col %	1<br>0.4<br>12.5	237<br>99.6<br>19.8	238<br>100.0<br>19.8
**16**<br>Row %<br>Col %	3<br>1.5<br>37.5	196<br>98.5<br>16.4	199<br>100.0<br>16.5
**17**<br>Row %<br>Col %	0<br>0.0<br>0.0	155<br>100.0<br>13.0	155<br>100.0<br>12.9
**18**<br>Row %<br>Col %	1<br>0.8<br>12.5	130<br>99.2<br>10.9	131<br>100.0<br>10.9
**19**<br>Row %<br>Col %	1<br>1.8<br>12.5	55<br>98.2<br>4.6	56<br>100.0<br>4.7
**20**<br>Row %<br>Col %	0<br>0.0<br>0.0	28<br>100.0<br>2.3	28<br>100.0<br>2.3
**21**<br>Row %<br>Col %	0<br>0.0<br>0.0	25<br>100.0<br>2.1	25<br>100.0<br>2.1
**22**	0	12	12

Row %<br>Col %	0.0<br>0.0	100.0<br>1.0	100.0<br>1.0
**23**<br>Row %<br>Col %	0<br>0.0<br>0.0	11<br>100.0<br>0.9	11<br>100.0<br>0.9
**24**<br>Row %<br>Col %	0<br>0.0<br>0.0	6<br>100.0<br>0.5	6<br>100.0<br>0.5
**25**<br>Row %<br>Col %	0<br>0.0<br>0.0	10<br>100.0<br>0.8	10<br>100.0<br>0.8
**26**<br>Row %<br>Col %	0<br>0.0<br>0.0	7<br>100.0<br>0.6	7<br>100.0<br>0.6
**27**<br>Row %<br>Col %	0<br>0.0<br>0.0	3<br>100.0<br>0.3	3<br>100.0<br>0.2
**32**<br>Row %<br>Col %	0<br>0.0<br>0.0	2<br>100.0<br>0.2	2<br>100.0<br>0.2
**33**<br>Row %<br>Col %	0<br>0.0<br>0.0	1<br>100.0<br>0.1	1<br>100.0<br>0.1
**TOTAL**<br>Row %<br>Col %	8<br>0.7<br>100.0	1196<br>99.3<br>100.0	1204<br>100.0<br>100.0

Descriptive Statistics for Each Value of Crosstab Variable

	Obs	Total	Mean	Variance	StdDev
Yes	8	127.0000	15.8750	3.8393	1.9594
No	1196	19325.0000	16.1580	7.4721	2.7335

	Minimum	25%	Median	75%	Maximum	Mode
Yes	13.0000	14.5000	16.0000	17.0000	19.0000	16.0000
No	7.0000	14.0000	16.0000	17.0000	33.0000	15.0000

ANOVA, a Parametric Test for Inequality of Population Means

(For normally distributed data only)

Variation	SS	df	MS	F statistic
Between	0.6366	1	0.6366	0.0854
Within	8956.0079	1202	7.4509	
Total	8956.6445	1203		

T Statistic = 0.2923

P-value = 0.7701

Bartlett's Test for Inequality of Population Variances

Bartlett's chi square= 1.1961 df=1 P value=0.2741

A small p-value (e.g., less than 0.05 suggests that the variances are not homogeneous and that the ANOVA may not be appropriate.

Mann-Whitney/Wilcoxon Two-Sample Test (Kruskal-Wallis test for two groups)

Kruskal-Wallis H (equivalent to Chi square) = .0019
Degreesoffreedom = 1
P value = 0.9651

PreviousDataset Results Library

FREQ HIVSifilis

Next Procedure

Forward

HIVSifilis	Frequency	Percent	Cum Percent	
**0**	1138	94.5%	94.5%	
**1**	62	5.1%	99.7%	
**2**	4	0.3%	100.0%	
**Total**	1204	100.0%	100.0%	

95% ConfLimits

0 93.0% 95.7%

1 4.0% 6.6%

2 0.1% 0.9%

[PreviousDataset](#) [Results Library](#)

TABLES SEXARCA HTLVHUPE

[Next Procedure](#)

[Forward](#)

	HTLV		
**SEXARCA**	**Yes**	**No**	**TOTAL**
**1**	3	555	558
Row %	0.5	99.5	100.0
Col %	37.5	46.4	46.3
**2**	5	641	646
Row %	0.8	99.2	100.0
Col %	62.5	53.6	53.7
**TOTAL**	8	1196	1204
Row %	0.7	99.3	100.0
Col %	100.0	100.0	100.0

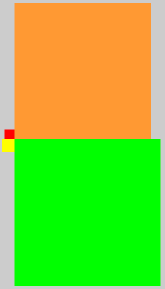

Single TableAnalysis

	Point	95% ConfidenceInterval	
	Estimate	Lower	Upper
PARAMETERS: Odds-based			

OddsRatio (crossproduct)	0.6930	0.1649	2.9128	(T)
OddsRatio (MLE)	0.6932	0.1359	3.0142	(M)
		0.1072	3.5811	(F)
PARAMETERS: Risk-based				
RiskRatio (RR)	0.6946	0.1667	2.8936	(T)
RiskDifference (RD%)	-0.2364	-1.1446	0.6719	(T)

(T=Taylor series; C=Cornfield; M=Mid-P; F=Fisher Exact)

STATISTICAL TESTS	Chi-square	1-tailed p	2-tailed p
Chi-square - uncorrected	0.2534		0.6146870377
Chi-square - Mantel-Haenszel	0.2532		0.6148340246
Chi-square - corrected (Yates)	0.0218		0.8825719344
Mid-p exact		0.3212220159	
Fisher exact		0.4454602675	

Warning: The expected value of a cell is <5. Fisher Exact Test should be used.

Previous Dataset Results Library

TABLES USOPRESERVANTESGRAV HTLVHUPE

Next Procedure

[Forward](#)

HTLV			
**USO DE PRESERVATIVO ANTES DA GRAVIDEZ**	**Yes**	**No**	**TOTAL**
**1**	3	718	721
Row %	0.4	99.6	100.0
Col %	37.5	60.1	59.9
**2**	5	477	482
Row %	1.0	99.0	100.0
Col %	62.5	39.9	40.1
**TOTAL**	8	1195	1203
Row %	0.7	99.3	100.0
Col %	100.0	100.0	100.0

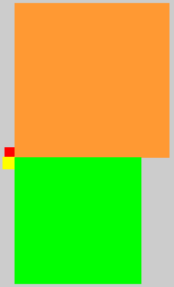

Single TableAnalysis

	Point	95% ConfidenceInterval		
	Estimate	Lower	Upper	
PARAMETERS: Odds-based				
OddsRatio (crossproduct)	0.3986	0.0948	1.6758	(T)
OddsRatio (MLE)	0.3989	0.0782	1.7349	(M)
		0.0617	2.0613	(F)
PARAMETERS: Risk-based				
RiskRatio (RR)	0.4011	0.0963	1.6706	(T)
RiskDifference (RD%)	-0.6213	-1.6406	0.3980	(T)

(T=Taylor series; C=Cornfield; M=Mid-P; F=Fisher Exact)

STATISTICAL TESTS	Chi-square	1-tailed p	2-tailed p
Chi-square - uncorrected	1.6878		0.1938871786
Chi-square - Mantel-Haenszel	1.6864		0.1940725500
Chi-square - corrected (Yates)	0.8784		0.3486470273
Mid-p exact		0.1117892291	
Fisher exact		0.1739270137	

Warning: The expected value of a cell is <5. Fisher Exact Test should be used.

TABLES NUMPARCSEXVIDA1 HTLVHUPE

HTLV			
**NÚM DE PARCEIROS ATÉ O MOMENTO**	**Yes**	**No**	**TOTAL**
**1**	4	789	793
Row %	0.5	99.5	100.0
Col %	50.0	69.6	69.4

**2**<br>Row %<br>Col %	4<br>1.1<br>50.0	345<br>98.9<br>30.4	349<br>100.0<br>30.6
**TOTAL**<br>Row %<br>Col %	8<br>0.7<br>100.0	1134<br>99.3<br>100.0	1142<br>100.0<br>100.0

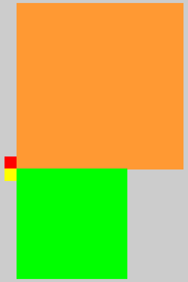

Single TableAnalysis

	Point Estimate	95% ConfidenceInterval Lower	Upper	
PARAMETERS: Odds-based				
OddsRatio (crossproduct)	0.4373	0.1087	1.7585	(T)
OddsRatio (MLE)	0.4376	0.0982	1.9503	(M)
		0.0810	2.3637	(F)
PARAMETERS: Risk-based				
RiskRatio (RR)	0.4401	0.1107	1.7497	(T)
RiskDifference (RD%)	-0.6417	-1.8625	0.5790	(T)

(T=Taylor series; C=Cornfield; M=Mid-P; F=Fisher Exact)

STATISTICAL TESTS	Chi-square	1-tailed p	2-tailed p
Chi-square – uncorrected	1.4347		0.2310055846
Chi-square - Mantel-Haenszel	1.4334		0.2312099074

Chi-square - corrected (Yates)	0.6604		0.4164025563
Mid-p exact		0.1327324923	
Fisher exact		0.2036940253	

Warning: The expected value of a cell is <5. Fisher Exact Test should be used.

Previous Dataset Results Library

TABLES ESCOLARIDADE HTLVHUPE

Next Procedure

Forward

	HTLV		
**ESCOLARIDADE**	**Yes**	**No**	**TOTAL**
**1**	3	425	428
Row %	0.7	99.3	100.0
Col %	37.5	35.7	35.7
**2**	5	767	772
Row %	0.6	99.4	100.0
Col %	62.5	64.3	64.3
**TOTAL**	8	1192	1200
Row %	0.7	99.3	100.0
Col %	100.0	100.0	100.0

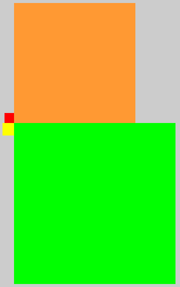

Single TableAnalysis

	Point Estimate	95% ConfidenceInterval Lower	Upper	
PARAMETERS: Odds-based				
OddsRatio (crossproduct)	1.0828	0.2575	4.5533	(T)
OddsRatio (MLE)	1.0828	0.2122	4.7100	(M)
		0.1673	5.5961	(F)
PARAMETERS: Risk-based				
RiskRatio (RR)	1.0822	0.2599	4.5066	(T)
RiskDifference (RD%)	0.0533	-0.9188	1.0253	(T)

(T=Taylor series; C=Cornfield; M=Mid-P; F=Fisher Exact)

STATISTICAL TESTS	Chi-square	1-tailed p	2-tailed p
Chi-square – uncorrected	0.0118		0.9135079980
Chi-square - Mantel-Haenszel	0.0118		0.9135439024
Chi-square - corrected (Yates)	0.0685		0.7935812742
Mid-p exact		0.4480137338	
Fisher exact		0.5884870985	

Warning: The expected value of a cell is <5. Fisher Exact Test should be used.

Previous Dataset Results Library

TABLES ESTADOCONJUGAL HTLVHUPE

Next Procedure

Forward

	HTLV		
**ESTADO CONJUGAL**	**Yes**	**No**	**TOTAL**
**1**	5	883	888
Row %	0.6	99.4	100.0
Col %	62.5	73.9	73.8
**2**	3	312	315
Row %	1.0	99.0	100.0
Col %	37.5	26.1	26.2
**TOTAL**	8	1195	1203
Row %	0.7	99.3	100.0
Col %	100.0	100.0	100.0

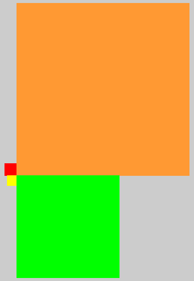

Single TableAnalysis

	Point Estimate	95% ConfidenceInterval Lower	Upper
PARAMETERS: Odds-based			
OddsRatio (crossproduct)	0.5889	0.1399	2.4786 (T)

OddsRatio (MLE)	0.5892	0.1353	3.0094	(M)
		0.1139	3.8159	(F)
PARAMETERS: Risk-based				
RiskRatio (RR)	0.5912	0.1421	2.4597	(T)
RiskDifference (RD%)	-0.3893	-1.5694	0.7908	(T)

(T=Taylor series; C=Cornfield; M=Mid-P; F=Fisher Exact)

STATISTICAL TESTS	Chi-square	1-tailed p	2-tailed p
Chi-square – uncorrected	0.5335		0.4651368652
Chi-square - Mantel-Haenszel	0.5331		0.4653224331
Chi-square - corrected (Yates)	0.1069		0.7436858526
Mid-p exact		0.2405651116	
Fisher exact		0.3510016370	

Warning: The expected value of a cell is <5. Fisher Exact Test should be used.

Previous Dataset Results Library

TABLES Gesta2 HTLVHUPE

Next Procedure

[Forward]

HTLV			
**Quantas vezes (total) já ficou grávida?**	**Yes**	**No**	**TOTAL**
**1**	3	728	731
Row %	0.4	99.6	100.0
Col %	37.5	69.7	69.4
**2**	5	317	322
Row %	1.6	98.4	100.0
Col %	62.5	30.3	30.6
**TOTAL**	8	1045	1053
Row %	0.8	99.2	100.0
Col %	100.0	100.0	100.0

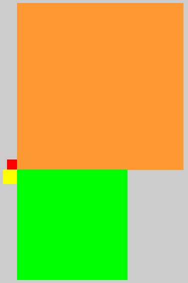

Single TableAnalysis

	Point	95% ConfidenceInterval		
	Estimate	Lower	Upper	
PARAMETERS: Odds-based				
OddsRatio (crossproduct)	0.2613	0.0621	1.0999	(T)
OddsRatio (MLE)	0.2616	0.0512	1.1394	(M)
		0.0404	1.3540	(F)
PARAMETERS: Risk-based				
RiskRatio (RR)	0.2643	0.0635	1.0993	(T)
RiskDifference (RD%)	-1.1424	-2.5702	0.2854	(T)

(T=Taylor series; C=Cornfield; M=Mid-P; F=Fisher Exact)

STATISTICAL TESTS	Chi-square	1-tailed p	2-tailed p
Chi-square - uncorrected	3.8693		0.0491786497
Chi-square - Mantel-Haenszel	3.8656		0.0492864437
Chi-square - corrected (Yates)	2.5024		0.1136721796
Mid-p exact		0.0370367018	
Fisher exact		0.0618682169	

Warning: The expected value of a cell is <5. Fisher Exact Test should be used.

Previous Dataset Results Library

TABLES ETNIA HTLVHUPE

Next Procedure

Forward

	HTLV		
**ETNIA / COR**	**Yes**	**No**	**TOTAL**
**1**	1	351	352
Row %	0.3	99.7	100.0
Col %	12.5	29.4	29.3

**2**	7	841	848
Row %	0.8	99.2	100.0
Col %	87.5	70.6	70.7
**TOTAL**	8	1192	1200
Row %	0.7	99.3	100.0
Col %	100.0	100.0	100.0

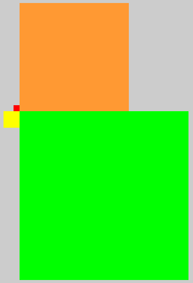

Single TableAnalysis

	Point	95% ConfidenceInterval		
	Estimate	Lower	Upper	
PARAMETERS: Odds-based				
OddsRatio (crossproduct)	0.3423	0.0420	2.7925	(T)
OddsRatio (MLE)	0.3425	0.0150	2.2268	(M)
		0.0076	2.6820	(F)
PARAMETERS: Risk-based				
RiskRatio (RR)	0.3442	0.0425	2.7870	(T)
RiskDifference (RD%)	-0.5414	-1.3660	0.2833	(T)

(T=Taylor series; C=Cornfield; M=Mid-P; F=Fisher Exact)

STATISTICAL TESTS	Chi-square	1-tailed p	2-tailed p
Chi-square - uncorrected	1.1009		0.2940632052
Chi-square - Mantel-Haenszel	1.1000		0.2942644545

Chi-square - corrected (Yates)	0.4352	0.5094611088
Mid-p exact		0.1646958476
Fisher exact		0.2678046753

Warning: The expected value of a cell is <5. Fisher Exact Test should be used.

Previous Dataset Results Library

TABLES RENDAFAMMENS1 HTLVHUPE

Next Procedure

Forward

	HTLV		
**RENDA FAMILIAR MENSAL ESTIMADA (EM SALÁRIOS MÍNIMOS)**	**Yes**	**No**	**TOTAL**
**1**	7	992	999
Row %	0.7	99.3	100.0
Col %	87.5	83.6	83.6
**2**	1	195	196
Row %	0.5	99.5	100.0
Col %	12.5	16.4	16.4
**TOTAL**	8	1187	1195
Row %	0.7	99.3	100.0
Col %	100.0	100.0	100.0

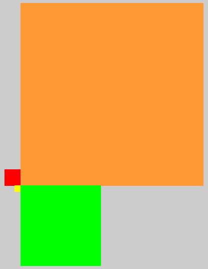

Single TableAnalysis

	Point Estimate	95% ConfidenceInterval Lower	Upper	
PARAMETERS: Odds-based				
OddsRatio (crossproduct)	1.3760	0.1683	11.2473	(T)
OddsRatio (MLE)	1.3757	0.2111	31.3986	(M)
		0.1751	62.3292	(F)
PARAMETERS: Risk-based				
RiskRatio (RR)	1.3734	0.1699	11.1006	(T)
RiskDifference (RD%)	0.1905	-0.9331	1.3141	(T)

(T=Taylor series; C=Cornfield; M=Mid-P; F=Fisher Exact)

STATISTICAL TESTS	Chi-square	1-tailed p	2-tailed p
Chi-square - uncorrected	0.0894		0.7649190638
Chi-square - Mantel-Haenszel	0.0893		0.7650145489
Chi-square - corrected (Yates)	0.0324		0.8571700900
Mid-p exact		0.4251148267	
Fisher exact		0.6127781285	

Warning: The expected value of a cell is <5. Fisher Exact Test should be used.

Previous Dataset Results Library

TABLES HIVSifilis HTLVHUPE

Next Procedure

Forward

HTLV			
**HIVSifilis**	**Yes**	**No**	**TOTAL**
**0**	7	1131	1138
Row %	0.6	99.4	100.0
Col %	87.5	94.6	94.5
**1**	1	65	66
Row %	1.5	98.5	100.0
Col %	12.5	5.4	5.5
**TOTAL**	8	1196	1204
Row %	0.7	99.3	100.0
Col %	100.0	100.0	100.0

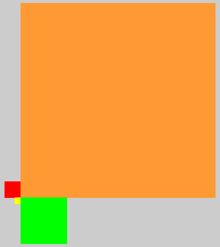

Single TableAnalysis

	Point Estimate	95% ConfidenceInterval Lower	Upper
PARAMETERS: Odds-based			
OddsRatio (crossproduct)	0.4023	0.0488	3.3189 (T)

OddsRatio (MLE)	0.4028	0.0610	9.2696	(M)
		0.0505	18.4069	(F)
PARAMETERS: Risk-based				
RiskRatio (RR)	0.4060	0.0507	3.2515	(T)
RiskDifference (RD%)	-0.9000	-3.8820	2.0819	(T)

(T=Taylor series; C=Cornfield; M=Mid-P; F=Fisher Exact)

STATISTICAL TESTS	Chi-square	1-tailed p	2-tailed p
Chi-square - uncorrected	0.7656		0.3815765974
Chi-square - Mantel-Haenszel	0.7650		0.3817743839
Chi-square - corrected (Yates)	0.0092		0.9236925101
Mid-p exact		0.2153977818	
Fisher exact		0.3638816425	

Warning: The expected value of a cell is <5. Fisher Exact Test should be used.

PreviousDataset Results Library

LOGISTIC HTLVHUPE = Gesta USOPRESERVANTESGRAV ESTADOCONJUGAL

Next Procedure

UnconditionalLogisticRegression

Term	OddsRatio	95%	C.I.	Coefficient	S. E.	Z-Statistic	P-Value
**Gesta**	3.6317	0.8522	15.4770	1.2897	0.7396	1.7437	0.0812
**USOPRESERVANTESGRAV**	2.4147	0.5643	10.3330	0.8816	0.7417	1.1886	0.2346
**ESTADOCONJUGAL**	1.9803	0.4633	8.4649	0.6832	0.7412	0.9218	0.3566
**CONSTANT**	*	*	*	-8.9838	2.0323	-4.4205	0.0000

**Convergence:**	Converged
**Iterations:**	6
**Final -2*Log-Likelihood:**	88.3735
**Cases included:**	1051

Test	Statistic	D.F.	P-Value
**Score**	6.0687	3	0.1083
**LikelihoodRatio**	5.6143	3	0.1320
